# Short- and long-term effects of beta-blockers on symptoms of anxiety and depression in patients with myocardial infarction and preserved left ventricular function: a pre-specified quality of life sub-study from the REDUCE-AMI trial

**DOI:** 10.1093/ehjacc/zuae112

**Published:** 2024-10-03

**Authors:** Philip Leissner, Katarina Mars, Sophia Humphries, Patric Karlström, Troels Yndigegn, Tomas Jernberg, Robin Hofmann, Claes Held, Erik M G Olsson

**Affiliations:** Department of Women’s and Children’s Health, Uppsala University, MTC-huset, Dag Hammarskjölds väg 14B, Akademiska sjukhuset, 752 37 Uppsala, Sweden; Department of Clinical Science and Education, Division of Cardiology, Karolinska Institutet, Södersjukhuset, Stockholm, Sweden; Department of Women’s and Children’s Health, Uppsala University, MTC-huset, Dag Hammarskjölds väg 14B, Akademiska sjukhuset, 752 37 Uppsala, Sweden; Department of Neurobiology, Care Science and Society, Karolinska Institutet, Stockholm, Sweden; Department of Internal Medicine, Ryhov County Hospital, Jönköping, Sweden; Department of Health, Medicine and Caring Sciences, Linköping University, Linköping, Sweden; Department of Cardiology, Clinical Sciences, Lund University and Skåne University Hospital, Lund, Sweden; Department Clinical Sciences, Danderyd Hospital, Karolinska Institutet, Stockholm, Sweden; Department of Clinical Science and Education, Division of Cardiology, Karolinska Institutet, Södersjukhuset, Stockholm, Sweden; Department of Medical Sciences, Cardiology, Uppsala Clinical Research Center, Uppsala University, Uppsala, Sweden; Department of Women’s and Children’s Health, Uppsala University, MTC-huset, Dag Hammarskjölds väg 14B, Akademiska sjukhuset, 752 37 Uppsala, Sweden

**Keywords:** Beta-blocker, Treatment, Anxiety, Depression, Linear mixed models, Psychological distress

## Abstract

**Aims:**

Among patients with myocardial infarction (MI) with preserved left ventricular ejection fraction (LVEF), the REDUCE-AMI trial did not demonstrate a benefit of beta-blocker vs. no beta-blocker treatment on all-cause mortality and recurrent myocardial infarction. The aim of this pre-specified sub-study was to investigate effects of beta-blockers on self-reported symptoms of anxiety and depression.

**Methods and results:**

In this parallel-group, open-label, registry-based randomized trial, assessments with the Hospital Anxiety and Depression Scale were obtained at hospitalization and two follow-up points (6–10 weeks and 12–14 months) after MI. Analyses were based on the intention-to-treat principle using linear mixed models, calculating both short- and long-term effects. From August 2018 through June 2022, 806 patients were enrolled. At baseline, 27% of patients were possible cases of anxiety (m, 5.6; SD, 3.9) and 14% were possible cases of depression (m, 3.9; SD, 3.2). Beta-blocker treatment had a negative effect on depressive symptoms at both follow-ups 1 (β = 0.48; 95% CI 09–0.86; *P* = 0.015) and 2 (β = 0.41; 95% CI = 0.01–0.81; *P* = 0.047), but no effect on anxiety.

**Conclusion:**

Beta-blocker treatment led to a modest increase in depressive symptoms among MI patients with preserved LVEF. This observed effect was most pronounced in individuals with prior beta-blocker treatment. In routine initiation and continuation of beta-blocker treatment, a risk of slightly increased depressive symptoms should be considered.

## Graphical Abstract

**Graphical Abstract:**
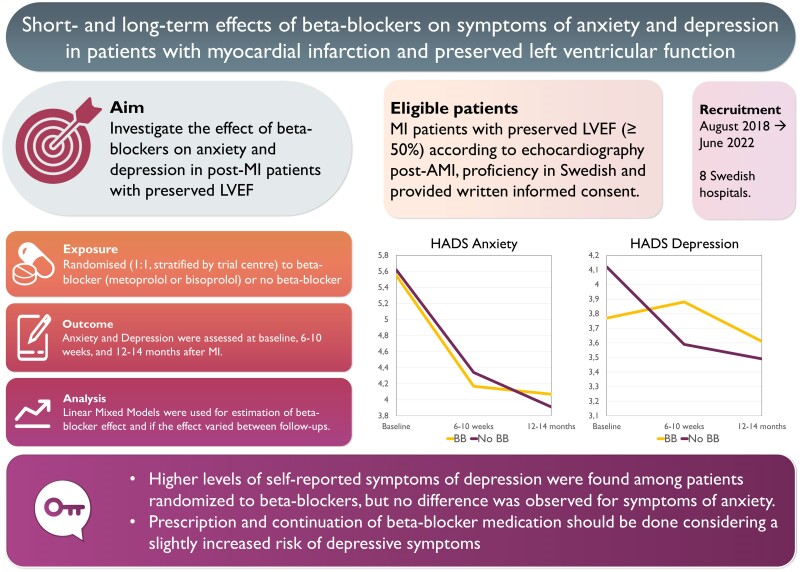

## Introduction

For decades, beta-blocker treatment has been a cornerstone in secondary prevention of patients following myocardial infarction (MI) based on seminal trials performed before the introduction of contemporary reperfusion treatment with percutaneous coronary intervention and antithrombotic agents.^1–3^ Beta-blockers suppress sympathetic tone through inhibition of adrenergic receptors intending to reduce myocardial oxygen demand, to prevent cardiac remodelling and arrhythmias.^4^ On the other hand, clinically relevant adverse effects of hypotension, bradycardia^5,6^ and more subjective symptoms like tiredness, exercise intolerance, and deterioration of psychosocial functioning due to nightmares, depression, and sexual dysfunction,^7–9^ have been reported, potentially affecting clinical benefits.

Earlier this year, the Randomized Evaluation of Decreased Usage of beta-bloCkErs (REDUCE-AMI) trial found no evidence of benefit of initiation of long-term treatment of beta-blockers in patients with acute MI and a preserved left ventricular ejection fraction (LVEF ≥ 50%) on subsequent cardiovascular endpoints.^10^ In contrast, the recently reported ABYSS (Assessment of Beta-Blocker Interruption 1 Year after an Uncomplicated Myocardial Infarction on Safety and Symptomatic Cardiac Events Requiring Hospitalization) trial did not find beta-blocker discontinuation to be non-inferior compared with continued treatment.^11^ Based on these controversial results, important clinical questions regarding routine initiation and discontinuation of beta-blockers in post-MI patients with preserved LVEF still remain. In this context, the patients’ mental well-being has to be taken into consideration. While depression is one of the most reported side effects of beta-blockers,^12,13^ previous studies have shown conflicting results,^13,14^ and randomized clinical trials (RCTs) employing active reporting and longer follow-ups have been called for.^15^ Furthermore, beta-blockers have previously been found effective in treating anxiety in psychiatric patients^16^ and in reducing emotional arousal during fear conditioning.^17^ Still, little is known of the effects in contemporary MI patients.

This exploratory, pre-specified, sub-study from the REDUCE-AMI trial aimed to investigate effects of long-term oral beta-blocker treatment in patients with MI and preserved LVEF on short- and long-term effects on self-reported levels of anxiety and depression, respectively.

## Methods

### Trial design and setting

This was a sub-study of the REDUCE-AMI (ClinicalTrials.gov identifier NCT03278509) trial,^18^ a prospective, open-label, parallel-group, registry-based RCT carried out in three countries: Sweden (38 centres), Estonia (one centre), and New Zealand (six centres). The design and rationale of the main trial and its primary results have been published previously.^10,18^ As reported, following considerations from the steering committee, the sponsor together with the steering committee, and patient representatives, the power calculation was adjusted aiming to detect a 25% relative risk reduction compared with the initially planned 17% in this event-driven trial.^10,18^ This change did not affect this sub-study in a negative way, as we are not reporting cardiovascular endpoints or mortality. On the contrary, recruitment in the present sub-study was longer than originally expected, and thus, more participants were enrolled.

Ethical approval was given by the Ethical Review Board in Stockholm (dnr 2016/1707-31/4 and 2018/1048-32). The current sub-study, REDUCE-Quality of Life (RQoL), was conducted at eight sites across Sweden and collected additional data on psychological distress, sexual dysfunction, and QoL measures, and the study design has been previously published.^19^ Here, we present data on self-reported levels of anxiety and depression.

### Participants and procedures

Potentially eligible RQoL participants were those randomized in the REDUCE-AMI trial,^10^ during the specified recruitment period.^19^ In summary, the REDUCE-AMI trial enrolled individuals meeting the following criteria: (i) age ≥ 18 years, (ii) recruited within 1–7 days after MI, (iii) underwent coronary angiography during hospitalization, (iv) confirmed obstructive coronary artery disease via coronary angiography, (v) preserved LVEF (≥50%) according to echocardiography post-AMI, and (vi) provided written informed consent. Exclusion criteria included (i) patients with conditions affecting their ability to adhere to the study protocol, (ii) contraindications for beta-blockers, or (iii) indication for beta-blockers other than as secondary prevention according to the treating physician.

At participating hospitals, patients randomized in the REDUCE-AMI study were also invited to participate in the RQoL sub-study. On top of the inclusion and exclusion criteria above, patients needed proficiency in the Swedish language. After additional written informed consent, patients were enrolled and could enter data online, via a secure Internet-based portal, or on paper. Data from self-reported questionnaires were collected at three time points: baseline (within 7 days of the MI), follow-up 1 (6–10 weeks after MI), and follow-up 2 (12–14 months after MI). Automatic reminders were sent to participants via e-mail and text message. If unresponsive, they were contacted by phone. Those who chose to fill out questionnaires by hand were mailed these in a pre-stamped envelope. Upon return, research staff entered the questionnaire responses manually into the Internet portal.

Measures on psychological distress, sexual dysfunction, and well-being were collected at all three time points. At baseline, additional data on physical activity, smoking, relationship status, education level, country of birth, preconceived notions about unwanted beta-blocker side effects, and psychotropic medication use were collected in a customized questionnaire. At follow-up, patients were asked about treatment adherence and an open question about possible experienced side effects.

### Sociodemographic and clinical characteristics

Data on age, sex, smoking status, beta-blocker medication status at discharge and at follow-ups, and clinical characteristics were retrieved from the Swedish Web System for Enhancement and Development of Evidence-based Care in Heart Disease Evaluated According to Recommended Therapies (SWEDEHEART) registry.^20^ Information on the date of death or emigration was obtained from the Swedish population registry.^21^ Physical activity was reported as how many days per week the patient engages in physical activity for at least 30 min that leads to elevated breathing and heart rate (0–7). Additional variables were categorical: *smoking* (never; previous; current), *psychotropic medication* (yes, if needed; yes, regularly; yes, both regularly and if needed; no), *education as highest finished level* (secondary school; high school; university/college studies up to 3 years; university/college studies longer than 3 years), *born in Sweden* (yes; no); *relationship status* (single; living with partner/married; living alone but in a relationship; other), and *belief about negative side effects of beta-blockers* (yes; unsure; no).

### Exposure

Patients who were randomly assigned to the beta-blocker group were administered metoprolol or bisoprolol during hospital stay and received a prescription for continued use after discharge. Responsible physicians were encouraged to prescribe at least 100 mg metoprolol or 5 mg bisoprolol per day.

### Outcome

The Hospital Anxiety and Depression Scale (HADS) is one of the most common tools for assessing anxiety and depression in a hospital setting.^22^ It has 14 items, with 7 focusing on symptoms of anxiety and 7 on symptoms of depression. The items are graded on a Likert-type scale and ranges from 0–3, the total score being 42. A higher score indicates a higher prevalence of symptoms, more symptoms, or both. A cut-off of >7 has been established for identifying possible cases of anxiety or depression,^23^ and the questionnaire has demonstrated good psychometric properties.^24^

### Randomization

Randomization in the main trial was stratified according to trial centre in a 1:1 ratio with permuted blocks and performed embedded in the SWEDEHEART registry.^25^ As the trial was open-label, group allocation was known by both participants and research staff.

### Sample size

Assuming a standardized mean difference of 0.25 for the outcome measure HADS, a sample size of 251 participants in each group would be enough to detect this difference with 80% power at a significance level of 5%. To increase power and to compensate for cross-over and loss-to follow-up, enrolment in the RQoL sub-study continued further beyond the initial target number of 502 individuals.

### Statistical analysis

The main analysis was based on the intention-to-treat principle. Descriptive statistics of continuous data are presented as mean and standard deviation (SD) and categorical and binary data as numbers (*N*) and %. Missing data were assumed to be missing at random (MAR) and was handled with multiple imputations, using chained equations and predictive mean matching.^26^ Imputation was stratified for the two treatment arms, and imputed values for individuals who died during the follow-up period were removed for follow-ups after the date of death. For the analysis excluding non-adherent participants, no imputation of beta-blocker status was made.

Data were structured as panel data, and linear mixed models were used to analyse the effect of beta-blocker assignment on anxiety and depression at follow-ups 1 and 2. The independent variable was beta-blocker group. Dependent variables consisted of the subscale scores of HADS. Separate regression models were fit for both main dependent variables. Additionally, to obtain more precise estimates, the following covariates were added to the model: baseline measures of the respective HADS subscale used in each analysis, education, born in Sweden, age, and sex. As the effect of beta-blocker medication may vary over time, an interaction term was added between the beta-blocker variable and the follow-up time point. As the effect of the baseline HADS values on outcome also likely varies over time, an additional interaction effect between the HADS baseline variable and the follow-up time point variable was added.

A stratified analysis was performed, using the same model specification as the main analysis and separating those who already had a beta-blocker prescription at randomization from those who did not. Additionally, two pre-specified sensitivity analyses^19^ and one *post hoc* sensitivity analysis were conducted. First, the main analysis was repeated without using multiple imputations, including only complete cases. Second, an analysis identical to the main analysis, but excluding those who did not adhere to their randomization condition according to the SWEDEHEART registry for secondary prevention, was conducted. Third, as there was a possibility that randomization status affected baseline values on HADS, all analyses were repeated without adjusting for baseline values. No correction for family-wise comparisons were made as the effect of beta-blockers on the two outcomes were investigated separately.^27,28^  *P*-values reported are two-sided, and 0.05 is held as the level of statistical significance. Stata Software Package (version 18.0) was used to perform all statistical analyses.

## Results

### Participants and baseline data

**Figure 1:**
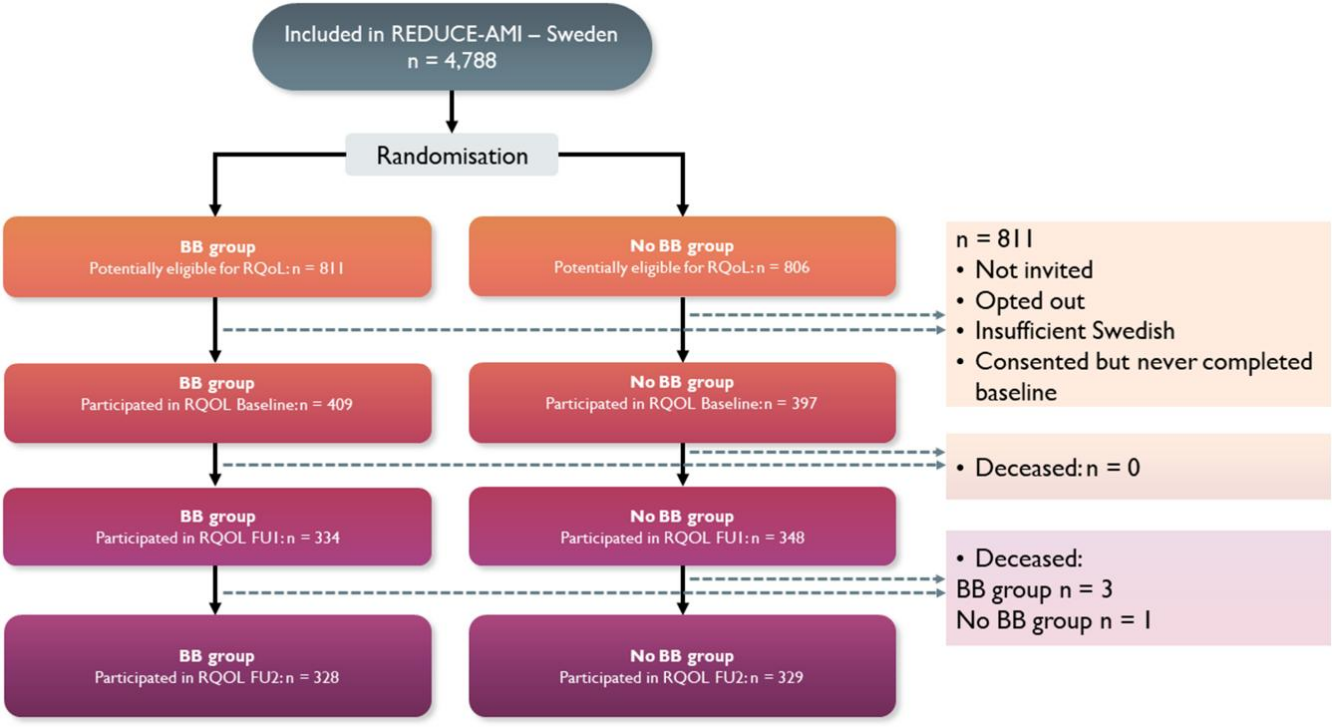
Flow chart of recruitment, randomization, and follow-up in REDUCE-Quality of Life.

A flowchart depicting the recruitment process is presented in *Figure 1*. Recruitment for RQoL started in August 2018 and ended in June 2022. Follow-up continued until June 2023. From a total of 1617 randomized, potentially eligible, main study participants, 806 were included in the RQoL sub-study (mean age 64.7 ± 10.4 years; 77% male). REDUCE-Quality of Life participants were on average younger and more often current smokers than the potentially eligible REDUCE-AMI participants that did not participate in RQoL but did not differ in respect to any other baseline characteristic (HADS—depression, *P* = 0.13; HADS—anxiety, *P* = 0.80). All baseline characteristics are presented in *Table 1*.

**Table 1:** Descriptive statistics

	REDUCE	RQoL	No BB	BB
	*N* = 811	*N* = 806	*N* = 397	*N* = 409
Age, mean (SD)	63.3 (11.2)	64.7 (10.4)	64.2 (10.1)	65.2 (10.7)
Male, % (*n*)	76% (618)	77% (624)	79% (312)	76% (312)
Hypertension, % (*n*)	48% (393)	50% (400)	49% (195)	50% (205)
Previous diagnosis of diabetes, % (*n*)	16% (131)	16% (128)	14% (57)	17% (71)
Previous PCI, % (*n*)	6% (45)	7% (58)	7% (29)	7% (29)
Previous stroke, % (*n*)	4% (29)	2% (18)	2% (8)	2% (10)
Previous myocardial infarction, % (*n*)	7% (53)	8% (63)	8% (30)	8% (33)
Type of beta-blocker medication, % (*n*)				
Bisoprolol	15% (122)	17% (136)		
Metoprolol	35% (280)	34% (273)		
Prior beta-blocker prescription^a^, % (*n*)	13% (106)	14% (111)	14% (56)	13% (55)
Systolic blood pressure, mean (SD)	151.1 (26.3)	151.2 (26.0)	151.5 (26.3)	150.8 (25.8)
Beta-blocker medication at FU1^g^	39% (315)	42% (336)	10% (40)	72% (296)
Beta-blocker medication at FU2^h^	37% (301)	39% (314)	13% (50)	65% (264)
Heart rate, mean (SD)	75.0 (15.1)	74.9 (15.5)	74.6 (16.1)	75.2 (15.0)
Current smoker^b^*, % (*n*)	21% (170)	16% (132)	19% (75)	14% (57)
Level of education^c^, % (*n*)				
Secondary school		21% (172)	20% (79)	23% (93)
High school		42% (341)	43% (170)	42% (171)
Undergraduate studies ≤ 3 years		19% (153)	19% (75)	19% (78)
Undergraduate studies > 3 years		17% (138)	18% (71)	16% (67)
In a relationship, % (*n*)		80% (644)	79% (315)	80% (329)
Born in Sweden, % (*n*)		89% (715)	88% (351)	89% (364)
Physically active at baseline^d^, % (*n*)		69% (556)	70% (276)	68% (280)
Psychotropic medication for anxiety or depression^e^		10% (78)	10% (38)	10% (40)
Expectation of beta-blocker adverse effects^f^**				
Yes		14% (110)	18% (72)	9% (38)
Unsure		61% (490)	62% (245)	60% (245)
No		17% (133)	11% (44)	22% (89)
HADS—anxiety^g^, mean (SD)		5.6 (3.9)	5.6 (4.0)	5.6 (3.8)
HADS—depression^g^, mean (SD)		3.9 (3.2)	4.1 (3.4)	3.8 (3.0)
Possible cases of anxiety, by HADS^i^		27% (115)	26% (103)	28% (115)
Possible cases of depression, by HADS^i^		14% (218)	15% (60)	13% (55)

REDUCE vs. RQoL comparison refers to the comparison between RQoL participants and REDUCE participants that were potentially eligible to participate in the RQoL study but did not. Possible cases defined as >7 score on respective HADS subscale.

Number of missing in RQoL data set: ^a^1, ^b^10, ^c^2, ^d^8, ^e^5, ^f^73, ^g^152, ^h^141, ^i^20.

PCI, percutaneous coronary intervention; HADS, Hospital Anxiety and Depression Scale; BB, beta-blocker; FU, follow-up.

^*^ *P* < 0.05, comparison REDUCE-AMI vs. RQoL.

^**^ *P* < 0.05, comparison BB vs. no BB in RQoL.

Out of the 806 RQoL participants, 682 (85%) participated in follow-up 1 and 657 (82%) participated in follow-up 2. Those who did not participate in the follow-ups were at baseline; younger, had more often diabetes, were current smokers, had lower education status, were born outside Sweden, were physically inactive, were taking psychotropic drugs, and had more anxiety and depression compared with those who participated in follow-ups. During the study period, four participants died between follow-up 1 and follow-up 2.

Regarding adherence to randomization group, at follow-up 1, 73 were non-adherent and 152 had unknown adherence status; at follow-up 2, 107 were non-adherent and 141 had unknown adherence status.

### Intention-to-treat analysis

**Figure 2:**
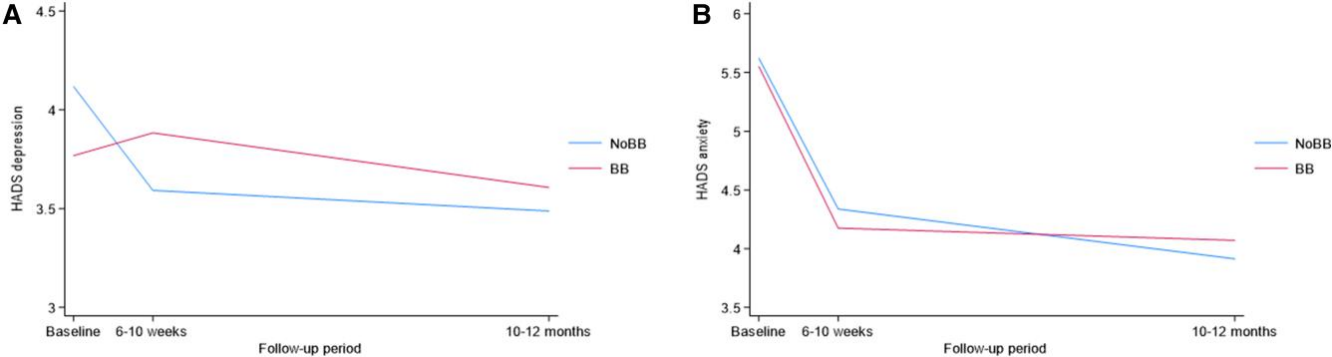
Effect of beta-blocker over time. (*A*) Hospital Anxiety and Depression Scale—depression; (*B*) Hospital Anxiety and Depression Scale—anxiety. BB, beta-blocker.

In the main analysis, the beta-blocker group had higher levels of depressive symptoms at both follow-ups, but no effect was observed on anxiety (*Table 2* and *Figure 2*).

**Table 2:** Regression coefficients if randomized to beta-blocker treatment

	2 months		12 months		Interaction^a^
	β (95% CI)	*P*-value	β (95% CI)	*P*-value	*P*-value
Main analysis (*N* = 806)					
HADS—Depression	0.48 (0.09, 0.86)	0.015	0.41 (0.01, 0.81)	0.047	0.744
HADS—Anxiety	−0.06 (−0.45, 0.34)	0.777	0.26 (−0.16, 0.68)	0.228	0.114
Sub-group, without beta-blocker at baseline (*N* = 694)					
HADS—Depression	0.41 (−0.00, 0.82)	0.050	0.27 (−0.16, 0.70)	0.222	0.516
HADS—Anxiety	−0.12 (−0.55, 0.31)	0.584	0.27 (−0.18, 0.73)	0.240	0.069
Sub-group, with beta-blocker at baseline (*N* = 111)					
HADS—depression	0.67 (−0.41, 1.75)	0.224	1.17 (0.03, 2.31)	0.044	0.363
HADS—anxiety	0.26 (−0.81, 1.33)	0.638	0.22 (−0.90, 1.34)	0.703	0.942

Adjusted for age, education, if born in Sweden, sex, and baseline assessment of outcome. Model includes interaction between variable for follow-up point and randomization group, as well as between follow-up point and baseline assessment of outcome. Data on prior registration of beta-blocker medication has one missing data point.

^a^Interaction between variable for follow-up point and randomization group.

### Treatment interaction effects with time and sex

In the main analysis, there was no interaction between beta-blocker and observation point for any of the outcomes (HADS—depression, *P* = 0.744; HADS—anxiety, *P* = 0.114). There was also no interaction between beta-blocker and sex for any of the outcomes (HADS—depression, *P* = 0.837; HADS anxiety, *P* = 0.351).

### Stratified analyses

**Figure 3:**
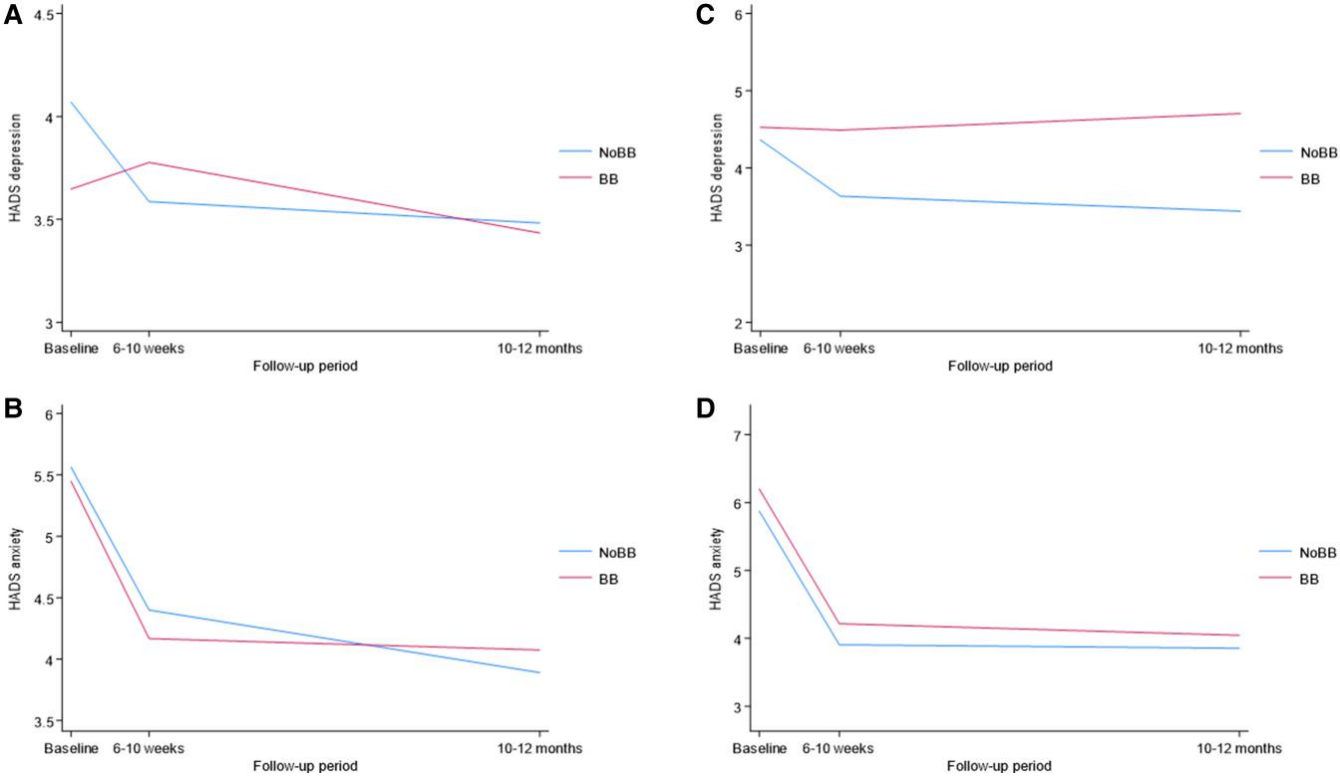
Effect of beta-blocker over time in strata. (*A*) Hospital Anxiety and Depression Scale—depression, no BB at baseline; (*B*) Hospital Anxiety and Depression Scale—anxiety, no BB at baseline; (C) Hospital Anxiety and Depression Scale—depression, BB at baseline; (D) Hospital Anxiety and Depression Scale—anxiety, BB at baseline. BB, beta-blocker.

Among patients without beta-blocker treatment prior to randomization (N = 694), those randomized to beta-blocker treatment had higher levels of depression at follow-up 1 (β = 0.41; 95% CI.00–0.82; *P* = 0.050), but not at follow-up 2 (*Table 2* and *Figure 3*). In the group of patients previously on beta-blocker treatment (*N* = 111), the beta-blocker group had higher levels of depression at follow-up 2 (β = 1.17; 95% CI.03–2.31; *P* = 0.044) but not at follow-up 1 (*Table 2* and *Figure 3*).

### Sensitivity analyses

Non-imputed analyses, only using complete cases, resulted in near identical results to the imputed, with no difference in interpretation.

The analysis including only adherent participants showed similar results except for the effect on depression at follow-up 1 that was no longer statistically significant and the effect size was smaller (β = 0.23; 95% CI −0.22 to 0.68; *P* = 0.316).

When repeating the main analysis without adjusting for baseline values, no difference between beta-blocker treated patients vs. those on no beta-blocker was found on either depressive or anxiety symptoms (*Table 3*). When repeating stratified analyses in the group who had beta-blocker treatment prior to randomization, without adjusting for baseline values, the beta-blocker group had numerically higher levels of depressive symptoms at follow-up 2 (β = 1.34; 95% CI −0.15 to 2.82; *P* = 0.078), although this was not statistically significant. No effect was observed for any other outcome or group (all *P*’s > 0.4).

**Table 3:** Main analysis, but without baseline values as covariates

	2 months		12 months		Interaction^a^
	β (95% CI)	*P*-value	β (95% CI)	*P*-value	*P*-value
HADS—depression	0.28 (−0.18, 0.74)	0.233	0.23 (−0.25, 0.71)	0.350	0.799
HADS—anxiety	−0.08 (−0.56, 0.40)	0.744	0.24 (−0.26, 0.74)	0.353	0.114

Adjusted for age, education, if born in Sweden and sex. Model includes interaction between variable for follow-up point and randomization group.

^a^Interaction between variable for follow-up point and randomization group.

## Discussion

In this pre-specified sub-study from the open-label REDUCE-AMI trial, MI patients with preserved LVEF randomized to initiation of long-term treatment with beta-blocker experienced higher levels of self-reported depressive symptoms at both follow-ups (6–10 weeks and 12–14 months post-MI), but no difference in symptoms of anxiety, than patients randomized to no beta-blocker use.

One of the proposed mechanisms in how beta-blockers may lead to increased depressive symptoms is through interaction with neurotransmitters.^29^ Aspects such as liposolubility, intrinsic sympathomimetic activity, and cardioselectivity may affect the autonomous nervous system (ANS) in different ways.^30^ Furthermore, beta-blockers may inhibit the level of engagement in joyful experiences (e.g. physical or sexual activities), which could lead to anhedonic symptoms of depression. As anhedonia has been proposed to be the depressive construct assessed with the HADS,^24,31^ our results may support this hypothesis.

In stratified analyses, we observed a difference between groups at the second follow-up, but not the first. Among those without beta-blockers before randomization, there was no evidence of increased depressive symptoms at either follow-up, but among those already on beta-blockers before randomization, evidence was found for an increase at follow-up 2, for the beta-blocker group. It is possible that the negative effects require a longer follow-up (>1 year) to be detected or that underlying differences between groups make patients react differently to the medication. In any case, discontinuation of beta-blockers could possibly alleviate existing effects and allow for a return to an improved psychological well-being.

A previous study suggested that the minimally clinically important difference of HADS—depression (range 0–21), in CVD patients, would be between 0.5 and 5.57 points.^32^ In our study, the absolute difference in depressive symptoms between the groups was relatively small (∼0.5 points), placing our results on the lower margin of, or perhaps even below, a clinically meaningful difference. The average difference in the stratum of patients on beta-blockers before randomization (∼1.2 points) suggests a slightly more significant difference, although still on the lower end.

Most previous studies have found beta-blockers effective in relieving symptoms of clinical anxiety in non-cardiac patients.^16,33^ In contrast, our study of MI patients did not find any support for an effect of beta-blockers on symptoms of anxiety, suggesting that these patients with subclinical levels of anxiety are not affected. Future research is needed to investigate if MI patients with preserved LVEF and clinical levels of anxiety are affected differently.

The findings of this sub-study should be considered in the ongoing debate regarding beta-blocker initiation and discontinuation for MI-patients with preserved LVEF. Currently, two major trials have been published investigating the effect on clinical outcomes; the REDUCE trial not showing evidence of benefit of initiation of beta-blockers on all-cause mortality and recurrent MI,^10^ whereas the ABYSS trial not demonstrating non-inferiority of beta-blocker discontinuation.^11^ Thus, the controversy of beta-blocker treatment post-MI is still highly relevant,^34–36^ and the ongoing REBOOT and BETAMI-DANBLOCK trials are expected to present their primary results soon.^37,38^

Regarding effects of beta-blockers on overall QoL, both REDUCE and ABYSS showed consistent neutral findings without any differential effect between the randomized groups.^11,39^ Both studies investigated QoL using the EQ-5D,^40^ which includes one item assessing anxiety/depression. As assessing both anxiety and depression in one item can be problematic both in regard to validity and reliability,^41^ it is a strength of the present study that we used separate scales assessing depression and anxiety from a specific, well-validated questionnaire.

Furthermore, while initiation of beta-blocker treatment showed no clear association with clinically relevant symptoms of anxiety or depression in our study, previous literature suggests that patients with less severe mental health issues need smaller changes to experience it as relevant.^32,42,43^ Furthermore, the 0.5 is an average effect, and some individuals may be more susceptible, possibly as indicated in the stratified analyses. Notwithstanding, even small changes may trigger a more severe disorder and might impact risk of future CV events.^44^ Consequently, we believe our results should be taken into consideration when prescribing beta-blocker therapy in MI patients with preserved LVEF. Regarding discontinuation of beta-blockers in previously treated MI patients, our findings are intriguing, but future RCTs designed specifically for this gap in evidence are needed.

Of note, several tests have been performed without adjusting for multiple comparisons. The main outcomes, anxiety and depression, while overlapping, are different and are hypothesized to be affected by beta-blockers by different mechanisms. Family-wise corrections in these cases can lead to biased conclusions.^27^ However, we are also aware that this can be debated. In unclear cases, it has been proposed that you present all *P*-values.^28^

### Limitations

In this open-label sub-study, measurements at baseline were completed after patients’ treatment assignment, which introduces a risk for bias. Although we could not find statistical justification for a difference between groups at baseline, repeating the main analysis without adjusting for baseline values showed substantial reductions in the effect on depressive symptoms. If the variation at baseline is due to the patients’ knowledge of randomization status, this adjustment introduces bias, but if the variation at baseline is random, adjusting for this will increase precision and power of the analysis. Additionally, some potentially eligible patients were not invited to the current sub-study. This was due to staffing irregularities that the authors of this current article cannot account for and serves as a potential source of selection bias. However, when comparing the REDUCE non-RQoL and RQoL participants they did not differ in most respects, only the RQoL participants more rarely being current smokers.

We also conducted an additional analysis, excluding non-adherent participants. Unfortunately, due to substantial amounts of missing data regarding beta-blocker status at follow-ups, inferences from this analysis are limited.

Furthermore, this sample of MI patients with preserved LVEF and no contraindications for beta-blockers may be a selected group. They are likely more healthy and appear to be less depressed than the typical MI patient, with around 14% possible cases of depression in our study, whereas the usual range is in between 20 and 40%.^45^ This should be considered when interpreting the results, and more studies are needed before generalizing to other contexts.

Power calculation was made based on using the full sample. Thus, the stratified analysis in patients on beta-blockers before randomization was not powered to detect differences on HADS and have a higher risk of a type 2 error. However, the largest effect sizes were observed in this stratum, and results were robust with and without baseline adjustments which can be hypothesis generating when designing definitive future studies. This limitation also applies for the comparison of potential clinical cases of anxiety and depression between the two groups. Given the low average reports of anxiety and depressive symptoms, these analyses would be underpowered. Future studies, with larger sample sizes are needed for conclusive evidence regarding this question.

The questionnaire used to assess anxiety and depression does not capture all aspects of these constructs. HADS is primarily focused on anhedonic symptoms of depression,^31^ and arousal symptoms of anxiety.^46^ Other symptoms, such as low mood and fatigue in depression and rumination, hypervigilance, and avoidance in anxiety, may be affected differently. This is important to consider when, for example, making associations with prognosis and mortality.^46,47^ Using a self-reported scale also has limitations, as an individual may be subject to bias and might misidentify symptoms that a medical professional would not. However, there are also advantages using self-reported scales, being easier to administer, sensitivity to smaller changes, and approximating the subjective experience.

## Conclusion

In this pre-specified sub-study from the REDUCE-AMI trial, MI patients with preserved LVEF randomized to initiation of long-term treatment with beta-blocker experienced slightly higher levels of self-reported depressive symptoms, but no difference in symptoms of anxiety, compared with patients randomized to no beta-blocker use. This effect may be especially pronounced among individuals with previous beta-blocker use. Given the remaining clinical controversy regarding the initiation and discontinuation of beta-blockers after AMI, a potential risk of slightly increased depressive symptoms found in our study should be considered.

## Data Availability

The data underlying this article cannot be shared publicly due to the General Data Protection Regulation (2016/679). The data will be shared on reasonable request to the corresponding author.
